# Health Principles

**Published:** 1873-10

**Authors:** 


### HEALTH PRINCIPLES.

A MAN had a finger nail torn off, causing very great pain; brown sugar was thrown on a pan of burning coals, and the finger held over the smoke for twenty minutes. The pain was removed, and in due time a cure was effected. In "Health at Home" it is narrated that a horse seemed to be dying of a festered wound. Some old shoes were cut up in a hog trough and set on fire under the horse, so that the smoke would reach the wound. In a few hours the swelling began to subside, the wound discharged, and the horse got well.

An old lady was knitting a stocking. A member of the family came in with a painful wound. She unravelled the stocking, put the yarn on a shovel of burning coals, caused the smoke to ascend against the wound, giving immediate relief.

The first thought of ordinary readers is that of wonder that such "simple" things should have such beneficial effects.

Instead of burdening the mind with the remembrance of old leather, and brown sugar, and yarn stockings, it is better to ascertain the general principles; for one may have the most agonizing sore, and be a thousand miles from an old shoe, or spoonful of brown sugar, or a yarn stocking. What then? In all cases there was smoke; out of smoke creosote is made, and carbolic acid is of the same essential nature; hence the application of these useful substances to all varieties of wounds, and burns, and sores. Their essential nature is twofold—they arrest decay and purify.

EAR ACHE

is said to be cured by roasting an onion and laying it on the part; a roasted frog, or the "entrails of a live chicken," or a flaxseed poultice would accomplish the same object. In all three cases the principle involved is warmth and moisture; for pain is the result of great cold or great heat, which latter is inflammation. Moisture causes evaporation, which carries off the extra heat. If the pain is the result of coldness, the warmth of a poultice, of whatever it may be made, is efficacious. In short, a skilful nurse can cure a great many aches and pains with water, hot or cold, according to circumstances. For example, the most agonizing pains resulting from

SPRAINS

are cured by allowing a stream of cold water to fall on the part continuously, until all pain is removed. The cold water carries off the heat, which is the inflammation, and causes the pain.

